# Co-existing phaeochromocytoma and anti-HMG-CoA reductase immune-mediated necrotising myositis: a diagnostic challenge

**DOI:** 10.1530/EDM-25-0052

**Published:** 2025-10-16

**Authors:** Sean Maher, Eibhlin Lonergan, Sarah Fullam, Brian O’Riordan, Ruth Prichard, Eric Heffernan, Sean Connolly, Carl Orr, Rachel K Crowley

**Affiliations:** ^1^ St Vincent’s University Hospital, Dublin, Ireland; ^2^ School of Medicine, University College Dublin, Dublin, Ireland

**Keywords:** adrenal, rare diseases/syndromes

## Abstract

**Summary:**

We report the case of a 62-year-old male who developed progressive lower limb and bulbar muscle weakness associated with elevated creatine kinase (CK) levels on a background of statin use following myocardial infarction. Electromyography, magnetic resonance imaging of thigh musculature, and muscle biopsy supported a diagnosis of necrotising myositis. Subsequently, antibodies to 3-hydroxy-3-methylglutaryl-CoA reductase were positive, confirming immune-mediated necrotising myositis (anti-HMGCR IMNM). An adrenal nodule detected on computed tomography of the thorax–abdomen and pelvis was confirmed to be a phaeochromocytoma following dedicated adrenal imaging and functional hormonal work-up, resulting in the second diagnosis. In-hospital treatment of the myositis consisted of intravenous immunoglobulin and methylprednisolone pulse therapy followed by high-dose oral steroids and mycophenolate therapy. He developed steroid-induced diabetes requiring insulin. After intensive rehabilitation and optimisation of blood pressure with alpha-blockade, he underwent a successful adrenalectomy for the phaeochromocytoma. Subsequently, immunosuppression was weaned, and the patient regained full muscle strength.

**Learning points:**

- Immune-mediated necrotising myopathy (IMNM) should be considered in patients with a history of statin use presenting with muscle weakness and elevated creatine kinase that fails to improve after statin cessation.
- Early diagnosis and immunosuppressive therapy are crucial in preventing permanent muscle damage. When IMNM is suspected clinically, work-up should include prompt testing for anti-HMGCR antibodies in serum.
- Rare conditions can co-exist, and multidisciplinary care in managing complex cases improves patient outcome.

## Background

Statin medications, as competitive inhibitors of the 3-hydroxy-3-methylglutaryl-CoA reductase (HMGCR) enzyme, reduce circulating concentrations of low-density lipoprotein (LDL). They lower cardiovascular risk as both primary and secondary prevention (1), and 2019 European Society for Cardiology guidelines advocate stringent LDL control (2). Statins can result in several muscle-related side effects due to myotoxicity (3). Autoantibodies can also target HMGCR, resulting in a subset of immune-mediated necrotising myopathy (IMNM) (3). Anti-HMGCR IMNM is a rare but severe condition causing proximal weakness, muscle fibre necrosis with minimal inflammatory cell infiltrate on muscle biopsy, and infrequent extra-muscular involvement (4). Although anti-HMGCR antibodies were first described in 2010 (5), there are sparse data on global incidence and whether it is increasing in line with statin prescription (6). A recent retrospective multisite cohort study across the UK and Australia found the mean annual incidence was 2.9 cases/million person-years (7). Interestingly, there was variability in dose and duration of treatment before disease onset and no clear relationship to the intensity of treatment. Statin-naïve IMNM, found in 7.5%, was more common in younger and non-white ethnicity. The John Hopkins myositis cohort (from 2002 to 2010) previously reported an incidence of two cases/million/year of anti-HMGCR IMNM (8). A 2018–2021 study from New Zealand exhibited a disease incidence of four cases per million per year; additionally, the incidence was approximately five times higher in the populations of Polynesian compared to those of European ancestry (9). Fenofibrate or ezetimibe monotherapy are the only other lipid-lowering therapies ever reported to induce such forms of myositis; however, there is a lack of data originating from clinical trials. A systematic review assessing the role of ezetimibe on myositis concluded that it does not appear to cause skeletal myositis (unlike statins), but this was hindered by the lack of large-scale trial data (10). Newer agents such as PCSK9 inhibitors have rarely caused such muscle symptoms, especially compared to statins and other non-statin treatment modalities (11). The National Lipid Association task force on lipid safety published a detailed clinical perspective on the management of statin-associated muscle symptoms (SAMS). While the paper does define myonecrosis under SAMS, it does not include immune-mediated myopathy and advocates against the routine measurement of anti-HMGCR antibodies. It recommends they may be ordered by a neurologist or a clinical lipid specialist for evaluation of persistent weakness, chronic creatine kinase elevations, or muscle pain or tenderness that does not remit with statin withdrawal (12).

Phaeochromocytoma, a rare tumour of the adrenal glands, produces excess catecholamines and can present with a variety of systemic symptoms, including myositis (13). It is postulated that catecholamine excess causes muscle cachexia due to hypermetabolism; this is particularly evident in patients with severe burns as a result of catecholamine surges (14). While other case reports have detailed phaeochromocytomas presenting with cardiomyopathy (15) and CNS symptoms with lower limb weakness (16), to the authors’ knowledge, this is the first to describe immune-mediated necrotising myopathy with a concomitant diagnosis of a phaeochromocytoma. The co-existence of these two rare conditions in the same patient presented a diagnostic and therapeutic challenge. This case emphasises the importance of a comprehensive and multidisciplinary approach to diagnosis and management of two rare conditions that can have severe sequelae if not identified promptly and treated appropriately.

## Case presentation

A 60-year-old male presented to the emergency department (ED) with a 2-week history of lower limb fatigue and decreased exercise tolerance. He was very active at baseline, cycling to work and coaching his son’s football team. On clinical examination, there was no upper or lower limb weakness. His serum creatine kinase (CK) was found to be elevated at 16,000 IU/L (normal range (NR): 40–320 IU/L); 24 h later, this was 11,000 IU/L after IV fluids. A connective tissue disease screen sent in ED was negative. Statin therapy was discontinued, and he was discharged to have urgent rheumatology follow-up. Over the ensuing week, he developed progressive muscle weakness, resulting in significant difficulty climbing stairs and dysphagia. He experienced no muscle pain or weight loss. There was no evident rash.

Medical history was significant for coeliac disease, stenosing tenosynovitis, and ST-elevation myocardial infarction (STEMI) 1 year previously. Medications included aspirin 75 mg, ramipril 2.5 mg, nebivolol 5 mg, atorvastatin 40 mg, and pantoprazole 40 mg. He denied palpitations, episodes of facial flushing, headaches, or diaphoresis. Physical examination at follow-up rheumatology clinic revealed symmetrical wasting of the thighs with reduced muscle strength in the lower limbs. Cardiovascular and respiratory examinations were unremarkable. The patient was admitted for further investigation.

## Investigation

Initial blood tests revealed: creatine kinase (CK) 17,532 IU/L (NR: 40–320 IU/L); aspartate aminotransferase (AST) 750 IU/L (NR: 11–34 IU/L); alanine aminotransferase (ALT) 1,011 IU/L (NR: 9–59 IU/L); troponin 356 ng/L (NR: 5–14 ng/L); and C-reactive protein (CRP) 7.8 mg/L (NR: 0–5 mg/L).

**Figure 1:**
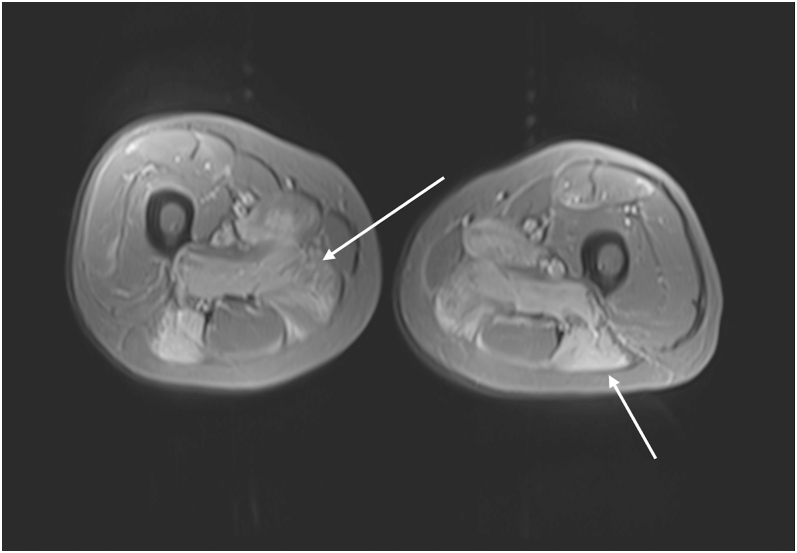
Axial short tau inversion recovery (STIR) MRI images of the lower limb. High signal intensity in the posterior and medial compartments of the thigh, in keeping with myositis. Imaging limited by motion artefact.

**Figure 2:**
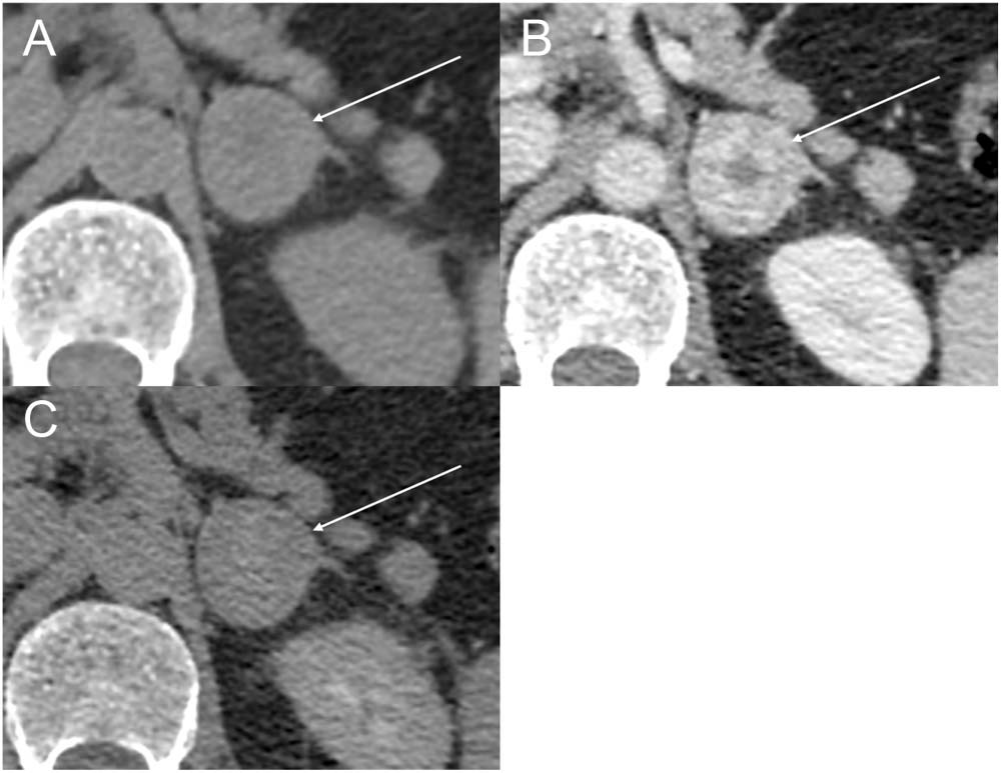
Axial CT images of a left 3.2 cm adrenocortical carcinoma. (A) Unenhanced CT demonstrates a lesion with a mean attenuation of 26 Hounsfield units (HU). (B) Contrast-enhanced CT at 75 s post-intravenous contrast administration shows heterogeneous enhancement, with a peak attenuation of 79 HU. (C) Delayed-phase CT at 10 min demonstrates near-complete washout, with an attenuation of 27 HU. The lesion’s heterogeneous enhancement and incomplete contrast washout are characteristic of malignant adrenal neoplasms, distinguishing it from benign adenomas.

Magnetic resonance imaging (MRI) showed a florid oedema signal pattern in the musculature of both thighs, consistent with myositis (Fig. 1). Echocardiogram was consistent with an old anterior myocardial infarction (MI). Oesophageal gastroduodenoscopy (OGD) revealed no structural abnormality. Computed tomography (CT) of the thorax–abdomen and pelvis identified a 3.1 cm left adrenal mass (Fig. 2). This raised the suspicion of paraneoplastic myositis, and this led to urgent functional hormonal work-up including AM cortisol, 24-h urinary cortisol, plasma metanephrines, renin, aldosterone, and adrenal androgens.

**Figure 3:**
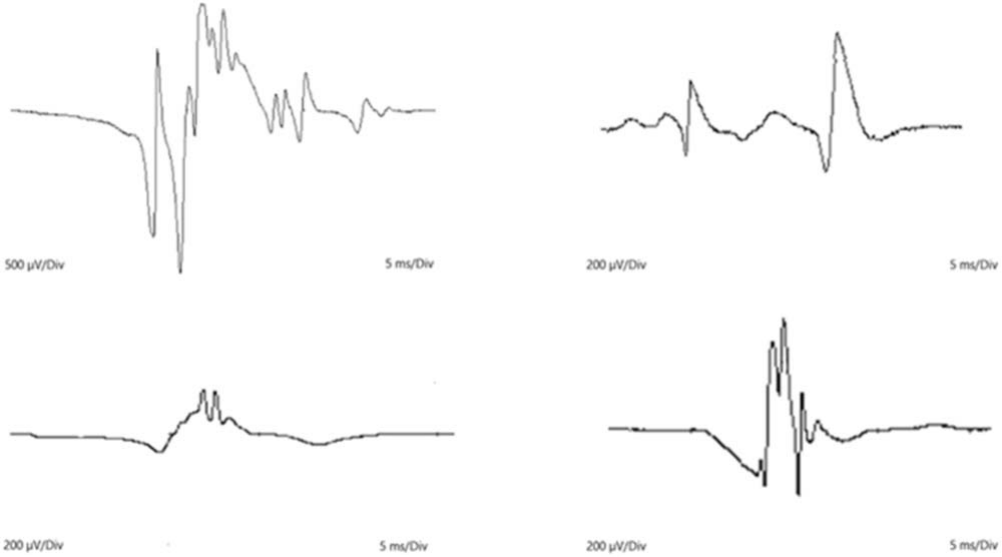
Low amplitude polyphasic motor unit potentials identified in (A) extensor digitorum communis, (B) deltoid, (C) vastus lateralis, and (D) tibialis anterior muscles.

Concentric needle electromyography (EMG) revealed fibrillations, positive sharp waves, and pronounced myotonic discharges at rest; low-amplitude polyphasic motor unit potentials (MUPs) were recruited. The findings were consistent with a severe, active necrotising myopathic process involving proximal and distal muscles in upper and lower limbs (Fig. 3). Histology of left biceps femoris muscle demonstrated a spotty myofibre necrosis pattern with upregulation of major histocompatibility complex class 1 (MHC1). Dedicated CT of adrenals and a functional adrenal hormone work-up showed plasma normetanephrines of 3,990 pmol/L (NR: <1,050), metanephrines of 1,160 pmol/L (NR: <360), and 3-methoxytyramine of 80 pmol/L. Aldosterone, renin, and adrenal androgens were within the reference range, as was 24-h urinary cortisol. One week later, anti-HMGCR antibody was strongly positive in serum at 363 CU (NR: <20). The remaining myositis screen was negative.

## Treatment

Atorvastatin was discontinued immediately on first presentation to ED. Intravenous immunoglobulins (IVIg) were administered to neutralise the assailant autoantibodies. The patient was commenced on IV methylprednisolone (1 g daily) and mycophenolate mofetil orally (500 mg twice daily) for immunosuppression of IMNM after urgent adrenal work-up ruled out Cushing’s syndrome. Subsequently, he was switched to oral prednisolone 80 mg/day and weaned appropriately. He developed hyperglycaemia and was found to have steroid-induced diabetes, leading to sulfonylurea and subcutaneous insulin therapy. He was commenced on alpha-blockade with doxazosin and later transitioned to phenoxybenzamine. Following intensive rehabilitation and blood pressure stabilisation, he underwent successful left-sided adrenalectomy.

## Outcome and follow-up

**Figure 4:**
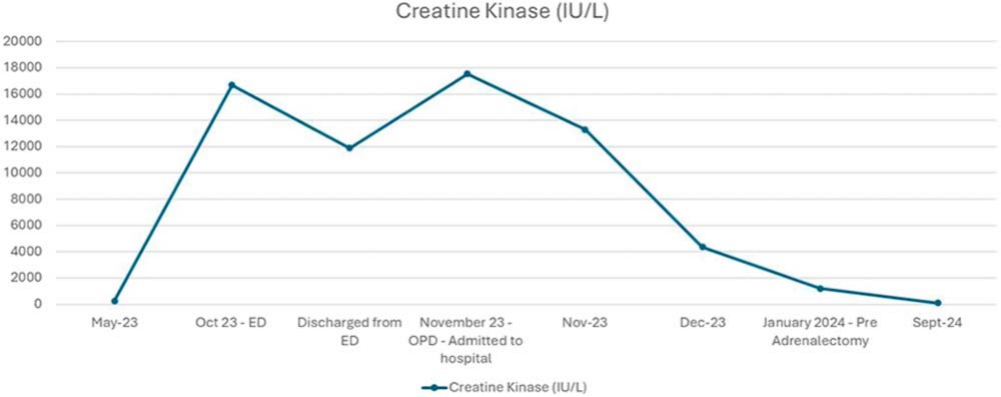
Line graph representing trends in creatine kinase (CK) levels.

The patient’s symptoms of muscle weakness began to improve with immunosuppressive therapy and rehabilitation, and CK levels decreased significantly (Fig. 4). He continued a tapering dose of prednisolone for IMNM. His post-operative outcome was excellent. Six months later, he had regained full muscle strength and returned to work.

## Discussion

This case illustrates the diagnostic challenges of managing co-existing immune-mediated necrotising myositis and phaeochromocytoma. The development of IMNM, a rare autoimmune condition, is particularly severe. The presence of anti-HMGCR antibodies provides a definitive diagnosis in such cases, and early treatment with immunosuppressants is crucial if suspected.

Simultaneously, the diagnosis of phaeochromocytoma complicated the clinical picture. The management of phaeochromocytoma requires careful preoperative alpha-blockade to prevent perioperative complications due to catecholamine excess. In this case, the presence of two concomitant diagnoses presented a further challenge for planning adrenalectomy, as the implications for general anaesthesia and recovery were unknown. A multidisciplinary approach involving endocrinology, rheumatology, radiology, clinical neurophysiology, neurology, neuropathology, and surgery was essential to successfully manage the overlapping diagnoses.

## Declaration of interest

The authors declare that there is no conflict of interest that could be perceived as prejudicing the impartiality of the work reported.

## Funding

This research did not receive any specific grant from any funding agency in the public, commercial, or not-for-profit sector.

## Patient consent

Written informed consent for publication of their clinical details and clinical images was obtained from the patient.

## Author contribution statement

SM was responsible for writing the manuscript and collating all clinical details. SF reviewed and edited the paper and provided clinical information on neurophysiology. BOR edited and provided radiological images. RC reviewed and edited the case report and provided clinical supervision as senior treating physician. CR, SC, RP, and EH all reviewed and approved the case report before submission.

## Patient’s perspective

The most surprising thing for me was that I experienced no pain, even though I had been feeling very weak before coming into the hospital. Upon admission, I underwent a series of tests, which was challenging, especially with the uncertainty of not knowing what was wrong or what the diagnosis might be. Throughout my stay, I received fantastic care and cannot thank the staff enough for their support. When I was informed that I had an adrenal tumour, it was a frightening moment, particularly because I had to wait to learn more about the type of tumour. However, I truly appreciated being kept informed throughout the process. Recovery has been slow, but I’m now back to playing nine holes of golf and doing aqua aerobics. While I don’t feel completely back to normal – maybe about 95% – I’m incredibly grateful to be alive and moving forward with my life.
